# Inflammation and pathological damage to the lungs of mice are only partially reversed following smoking cessation on subacute exposure to cigarette smoke

**DOI:** 10.3892/mmr.2015.3337

**Published:** 2015-02-11

**Authors:** HENGYI YAN, LI ZHAO, XIAOJIE WU, HONGBO LIU, CEN WU, YU LI, WEI ZHENG, HONGFANG JIANG

**Affiliations:** Department of First Respiratory Medicine, Shengjing Hospital of China Medical University, Shenyang, Liaoning 110004, P.R. China

**Keywords:** emphysema, airway inflammation, smoking cessation, transforming growth factor-β1, matrix metalloproteinase-12

## Abstract

The present study aimed to observe the level of inflammation and the number of lesions in the airways and parenchyma of mouse lungs subsequent to smoking cessation following 4 weeks exposure to cigarette smoke. Enlargement of the regional airspaces, deposition of peribronchial collagen fibers and macrophage infiltration were assessed. In addition, the expression levels of matrix metalloproteinase (MMP)-12 and transforming growth factor (TGF)-β1 were detected in the airways and lung parenchyma of C57BL/6 J mice. Mice, which were exposed to filtered air for 4 weeks or cigarette smoke for 8 weeks were used as control groups. A 4 week duration of smoke exposure induced the expansion of alveolar spaces ~100 *μ*m from the terminal bronchioles, but without increased deposition of collagen around the small airways, which was not reversed following smoking cessation. Pulmonary infiltration of macrophages and the protein expression levels of MMP-12 and TGF-β1 increased in the airways following 4 weeks smoke exposure, however, there was no further increase at 8 weeks, and the expression levels of TGF-β1 in the lung parenchyma decreased. At 4 weeks post-smoking cessation, the expression levels of TGF-β1 in the airways and lung parenchyma returned to normal; whereas, 1 week after smoking cessation, the expression levels of MMP-12 were higher compared with the normal control group. Subacute exposure to cigarette smoke induced an inflammatory response and regional damage to the lung parenchyma, prior to deposition of collagen around the airways. Following smoking cessation, the pulmonary inflammatory reaction was partially reversed, however, macrophage infiltration and the expression levels of MMP-12 remained significantly higher compared with the control mice. These results suggested that regulation of the expression of MMP-12 and TGF-β1, particularly in the distribution in the airways and lung parenchyma, may be a strategy for the early treatment of chronic obstructive pulmonary disease.

## Introduction

Chronic obstructive pulmonary disease (COPD) is characterized by the progressive obstruction of airflow, which is accompanied by an increased chronic inflammatory response in the airway and lung parenchyma (1). The inflammatory response is induced by deleterious particles or gases (1). Airway inflammation and remodeling induced by cigarette smoke, particularly the enlargement of alveolar airspaces and remodeling of small airways, are the pathological basis of airflow obstruction (2–4). However, the inflammatory response in the airway precedes typical pathological changes (5). Therefore, investigation of the cellular and molecular mechanisms underlying the early involvement of chronic smoking on the inflammatory response and its pathological damage to lung tissue is important for understanding the pathogenesis of COPD and for providing novel treatment strategies for early intervention.

Smoking cessation relieves airflow obstruction and the inflammatory response (6,7). However, previous human and animal experiments have demonstrated that, once COPD is initiated, the pulmonary inflammatory response continues (8–10) and the enlarged alveolar airspace cannot be reversed following smoking cessation (11). The outcome of collagen deposition in the walls of the small airways remains to be elucidated. Furthermore, the pathological and inflammatory outcomes in the lung following cessation in the early stages of chronic smoking, prior to the complete development of emphysema, remain unclear.

The present study observed the levels of inflammation and lesions in the lungs of mice exposed to cigarette smoke, and the changes following smoking cessation. The study also aimed to investigate the effects of early smoking cessation, and to examine the pathogenic mechanisms occurring at a stage when COPD may have an increased possibility of being reversed.

## Materials and methods

### Ethics

The present study was approved by the Ethics Committee of Shengjing First Affiliated Hospital of China Medical University (Shenyang, China).

### Animals and study procedures

A total of 25 (5 in each group) female specific pathogen-free (SPF) C57BL/6 J mice (8–12-weeks-old; 20.2±2.1 g) were purchased from the Experimental Animal Centre, Shengjing Hospital of China Medical University. The mice were bred in SPF conditions and were maintained at a constant temperature and humidity, with *ad libitum* access to food and water.

The mice were randomized into five groups. In the subacute smoking group, the mice were exposed to cigarette smoke for 4 weeks. In the 1-week smoking cessation group, the mice were exposed to cigarette smoke for 4 weeks followed by 1 week exposure to room air. In the 4-week smoking cessation group, the mice were exposed to cigarette smoke for 4 weeks followed by 4 week exposure to room air. In the normal control group, the mice were exposed to room air for 4 weeks. In the chronic smoking control group, the mice were exposed to cigarette smoke for 8 weeks (5). The mice were sacrificed within 24 h following the final exposure to air or smoke.

### Exposure to cigarette smoke

As described previously (12,13), exposure to cigarette smoke was performed in a chamber (160×50×50 cm; 20–24°C; 12-h light/dark cycle) attached to a cigarette-smoke generator, which was constructed for the present study by burning a cigarette in a sterile bottle, then blowing the smoke into the chamber with an electromagnetic air pump. The smoke from 10 sequentially ignited cigarettes (Red Dragon™; Wuhan Red Dragon Co. Ltd., Wuhan, China), each containing13 mg tar and 1.3 mg nicotine, and fresh air were introduced simultaneously into the chamber. Each session of cigarette smoke exposure lasted ~90 min, twice daily for five days each week (Monday to Friday).

### Histological and morphometric analyses

The mice (n=8), used for morphometric analyses were anesthetized with 10% chloral hydrate (0.03 ml/10 g) and sacrificed by exsanguination of the abdominal aorta. The lungs were isolated and fixed by infusion with 4% paraformaldehyde (Sinopharm Chemical Reagent Co., Ltd., Shanghai, China) through a tracheal cannula at a constant pressure of 25 cm H_2_O, part of the left lung was frozen and embedded in OCT Compound (#4583; Sakura Finetek USA, Inc., Torrance, CA, USA), and other parts were then immersed in paraformaldehyde. Between 24 and 48 h later, a mid-sagittal slice of the left lung was embedded in paraffin (Kangtai Clinical Reagent Co., Ltd., Beijing, China), and 5 *μ*m sections were dewaxed and hydrated prior to histological analyses. The sections were then stained with hematoxylin and eosin and Sirius Red (Sigma-Aldrich China, Inc., Shanghai, China) for detection of collagen fibers.

### Airspace enlargement

Airspace enlargement was evaluated, as previously described by Biselli *et al* (14). Briefly, the sections were placed under an Eclipse E800 light microscope (Nikon Corporation, Tokyo, Japan) connected to a R1 Wireless Close-Up Speedlight System video camera (Nikon Corporation) at x400 magnification. Images were captured and displayed on a monitor. A frame of five concentric semi-circles, with radii of 50, 100, 150, 200 and 250 *μ*m, was attached to the monitor screen with the center of the semi-circles placed over a terminal bronchiole, thereby allowing the semi-circle lines to lie over the lung parenchyma. The length of each of the five semi-circles was divided by the number of intersections between the line and the lung tissue. A total of 10 fields per slide were selected according to methods described by Biselli *et al* (14) then analyzed, producing one measurement of the degree of airspace enlargement at each of the five distances from the terminal bronchiole.

### Collagen measurement

The collagen in the airway walls was stained using Sirius Red and scored quantitatively. A ttoal of three lung sections in each mouse were examined. Referring to the method of Bracke *et al* (15), the length of the basement membrane (Pbm) and the area in the airway wall covered by the stain were determined using Image-Pro Plus 6.0 software (Media Cybernetics, Inc., Rockville, MD, USA). The area of collagen was normalized to Pbm. All airways with a Pbm <2,000 mm and cut at reasonable cross-sections, defined by a ratio of minimal-to-maximal internal diameter of 0.5, were included.

### Immunohistochemistry

For immunohistochemical analyses, the lung sections were deparaffinized and hydrated and endogenous peroxidase was blocked by 3% hydrogen peroxide (Jiutian Pharmaceutical Co., Ltd., Anshan, China). Antigen retrieval was performed as follows: Slices were dipped in 0.01 M citrate buffer (pH 6.0) and microwave heated to boiling for 10 min, then this was repeated twice further. The sections were then blocked with rabbit serum blocking reagent (Boster Biological Technology, Ltd., Wuhan, China) at room temperature for 20 min, and were incubated overnight at 4°C with rat anti-mouse Mac-3 monoclonal antibody (clone M3/84; 1:150 dilution; #553322; BD Biosciences, San Jose, CA, USA), or rabbit anti-mouse transforming growth factor (TGF)-β1 polyclonal antibody (1:100 dilution; sc-146; Santa Cruz Biotechnology, Inc., Dallas, TX, USA). The sections were then incubated with goat anti-rat (1:300 dilution; sc-2041) or goat anti-rabbit (1:300 dilution; sc-2040) biotin-conjugated secondary antibodies (Santa Cruz Biotechnology, Inc.) at 37°C for 2 h. Diaminobenzidine (Boster Biological Technology, Ltd.)was used as a chromogen, and the nuclei were stained with hematoxylin. Negative controls were obtained by omitting the primary antibody.

Analyses of alveolar macrophages (AMs) were performed by counting the number of cells and dividing this number by the tissue area in the same visual field. Analyses of the expression levels of TGF-β1 were performed in the airways and lung parenchyma. Images were captured of 10 fields of parenchyma and five airways in each mouse. The stained area was calculated using Image-Pro Plus software and divided by the tissue area.

### Western blotting

Lung tissues were homogenized on ice by cytoplasmic protein extraction using a Vibra-Cell VCX130 ultrasonic processor (Sonics & Materials, Inc., Newtown, CT, USA). Protein samples were extracted from lung-tissue homogenates using a Total Cytoplasm-Nuclei Protein Extract kit (Bio-Rad Laboratories, Inc., Hercules, CA, USA), according to the manufacturer’s instructions. The samples were normalized for total lung protein content using a BCA Protein Assay kit (Beyotime Institute of Biotechnology, Jiangsu, China) prior to further analyses. A total of 60 *μ*g protein samples were separated by 10% SDS-PAGE and transferred to polyvinylidene difluoride membranes (Beyotime Institute of Biotechnology). The membranes were then incubated for 2 h in a blocking solution (1X TBS with 5% defatted milk powder and 0.1% Tween-20), followed by incubation overnight at 4°C with rabbit anti-mouse matrix metalloproteinase (MMP)-12 polyclonal antibody (1:400 dilution; sc-30072) and rabbit anti-mouse β-actin polyclonal antibody (1:400 dilution; sc-130656) (Santa Cruz Biotechnology, Inc.). Following washing with Tris-buffered saline with 0.1% Tween-20 (TBST), the membranes were incubated with goat anti-rabbit horseradish peroxidase conjugated secondary antibodies (1:5,000 dilution; Santa Cruz Biotechnology, Inc.) for 2 h at room temperature. Each step was concluded by three rinses with TBST. BeyoECL Plus electrochemiluminescence substrate (Beyotime Institute of Biotechnology) was used for photographic development and fixation of the blots. Following light exposure, the membranes were chemically stripped to remove the antibodies and then reprocessed, but with antibodies targeting the internal control, β-actin. Images were captured and gray values calculated using Quantity One software, version 4.6.2 (Bio-Rad Laboratories, Inc.). The quantities of target protein were normalized to the value for β-actin in that sample.

### Immunofluorescence

To confirm the activation and expression of MMP-12 in the macrophages, double immunofluorescent staining was performed in the frozen lung sections. A combination of the rat anti-mouse Mac-3 and rabbit anti-mouse MMP-12 primary antibodies was used, and phosphate-buffered saline (PBS) was used as the negative control. The working dilutions of the Mac-3 and MMP-12 antibodies were 1:50 and 1:100, respectively. Working concentrations of goat anti-rat fluorescein isothiocyanate (FITC)-conjugated IgG (bsF-0293G) and goat anti-rabbit tetramethylrhodamine (TRITC)-conjugated IgG (bsf-0295G) (BIOSS, Beijing, China) were 1:100. The sections were incubated with primary antibodies at 4°C overnight, followed by incubation with the goat anti-rabbit horseradish peroxidase-conjugated secondary antibodies (1:5,000 dilution; sc-2004; Santa Cruz Biotechnology, Inc.) for 2 h at room temperature in the dark. Following primary and secondary antibody incubation, the sections were washed with PBS twice then incubated with 4′,6-diamidino-2-phenylindole dihydrochloride (DAPI; BIOSS) for 20 min at room temperature in the dark. Microscopic red fluorescence indicated the expression of MMP-12 antigen, labeled by TRITC; green fluorescence indicated macrophages, labeled by FITC; and blue fluorescence indicated DAPI-stained nuclei. Images of the three fluorescence channels were superimposed using Nikon EZ-C1 FreeViewer image analysis software, version 3.0 (Nikon Corporation), with yellow fluorescence indicating colocalization of the MMP-12 antigen and macrophages.

### Statistical analyses

Statistical analyses were performed using SPSS 11.5 (SPSS, Inc., Chicago, IL, USA). The data are presented as the mean ± standard error of the mean. The results were analyzed by one-way analysis of variance. A least significant difference *post hoc* test was used to determine significant differences between the groups. P<0.05 was considered to indicate a statistically significant difference.

## Results

### Enlargement of alveolar airspaces

The alveolar airspaces ~100 *μ*m from the terminal bronchioles were significantly enlarged in the mice exposed to cigarette smoke for 4 weeks compared with the control mice (P=0.004). This enlargement was not reversed following smoking cessation, however, exposure to cigarette smoke for 8 weeks did not result in further enlargement of the alveolar airspaces (Table I). Sizes of the alveolar airspaces ~50, 150, 200 and 250 *μ*m from the terminal bronchioles were not significantly different between the groups of mice.

### Collagen deposition around the small airways

**Figure 1:**
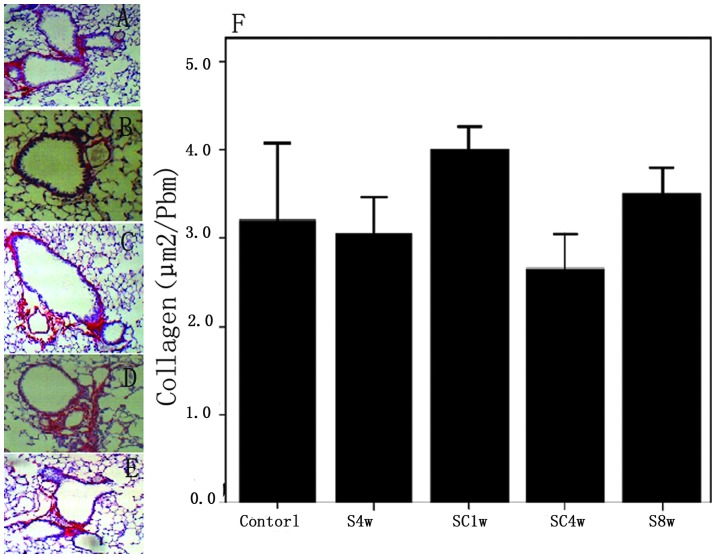
View of each group at 400x magnification. Red indicates staining of collagen by Sirius red. in the (A) control; (B) mice exposed to cigarette smoke for 4 weeks (S4w); (C) mice exposed to cigarette smoke for 8 weeks (S8w); (D) mice exposed to cigarette smoke for 4 weeks and room air for 1 week (SC1w); (E) mice exposed to cigarette smoke for 4 weeks and room air for 4 weeks (SC4w). (F) Analysis of collagen deposition around the small airways. The area of collagen was normalized to the length of the basement membrane (Pbm). No significant differences were detected between the groups. Data are presented as the mean ± standard error.

The deposition of collagen around the small airways was not significantly increased in the mice exposed to cigarette smoke for 4 or 8 weeks compared with the normal control group. In addition, no significant difference was observed in the collagen deposition prior to or following smoking cessation (Fig. 1).

### Macrophage infiltration

**Figure 2:**
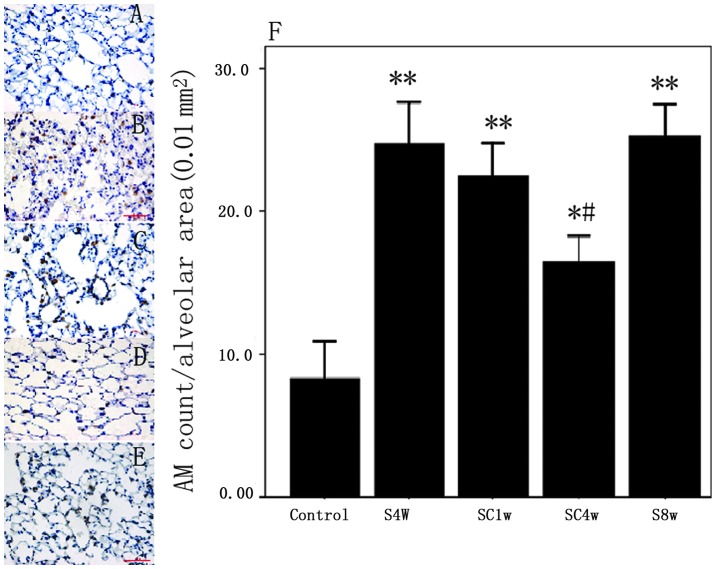
AM immunohistochemical staining from each group at 400x magnification. Brown was considered a positive result. (A) Control; (B) mice exposed to cigarette smoke for 4 weeks (S4w); (C) mice exposed to cigarette smoke for 8 weeks (S8w); (D) mice exposed to cigarette smoke for 4 weeks and room air for 1 week (SC1w); (E) mice exposed to cigarette smoke for 4 weeks and room air for 4 weeks (SC4w). (F) Analysis of number of macrophages per 0.01 mm^2^ alveolar area. Data are presented as the mean ± standard error. ^*^P<0.05 and ^**^P<0.001 vs. the control mice; ^#^P<0.05 vs. the S4w mice. AM, alveolar macrophage.

Macrophage infiltration into the lungs was significantly increased in the mice exposed to cigarette smoke for 4 weeks compared with the normal control group (P=0.001). The increased pulmonary infiltration of macrophages was significantly decreased after 4 weeks of smoking cessation (SC4w, vs. S4w; P=0.022); however, the levels did not return completely to the those observed in the normal control group (SC4w vs. control; P=0.015). Furthermore, mice exposed to cigarette smoke for 8 weeks did not exhibit increased macrophage infiltration compared with the mice exposed to cigarette smoke for 4 weeks (Fig. 2).

### Expression and distribution of MMP-12 protein

**Figure 3:**
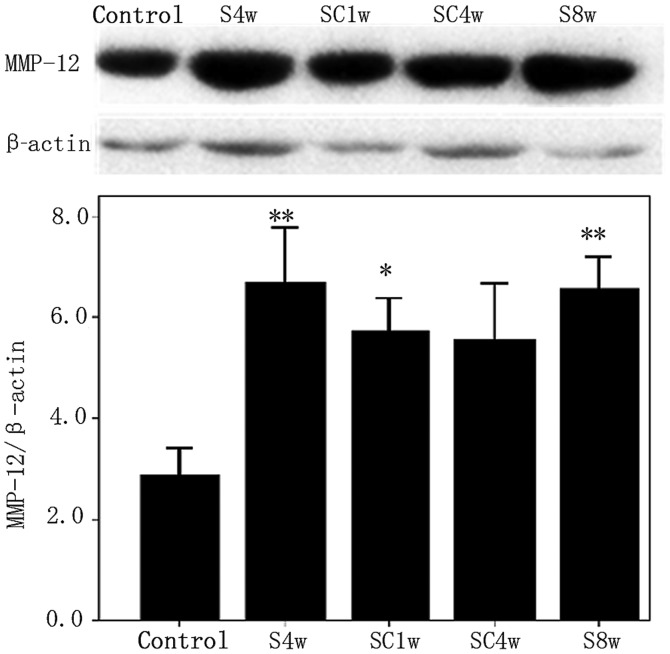
Western blot analysis of MMP-12 and β-actin protein expression. Comparison of the protein expression levels of MMP-12 between the various groups S4w, mice exposed to cigarette smoke for 4 weeks; SC1w, mice exposed to cigarette smoke for 4 weeks followed by 1 week exposure to room air; SC4w, mice exposed to cigarette smoke for 4 weeks followed by four weeks exposure to room air; S8w, mice exposed to cigarette smoke for 8 weeks. Data are presented as the mean ± standard error. ^*^P<0.05 and ^**^P<0.01 vs. the control mice. MMP, matrix metalloproteinase.

**Figure 4:**
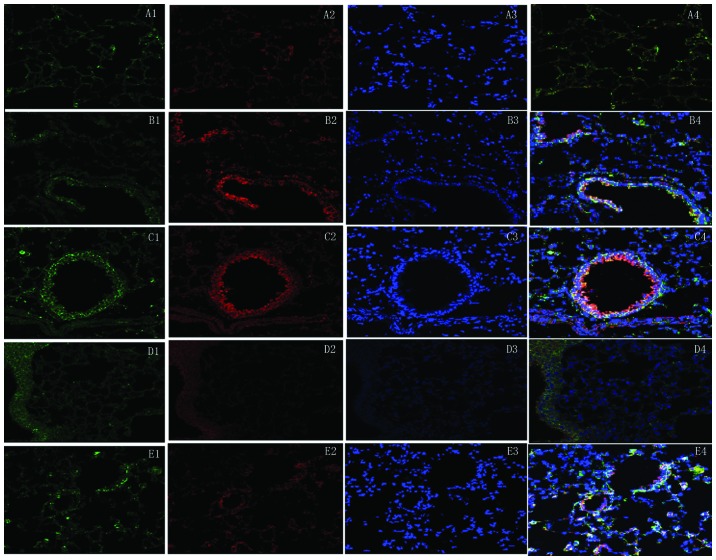
Double immunofluorescent staining for Mac-3 and MMP-12 in frozen lung sections at 400x magnification. Mac-3 staining indicates the positioning of macrophages in the (A) control, (B) mice exposed to cigarette smoke for 4 weeks (S4w); (C) mice exposed to cigarette smoke for 8 weeks (S8w); (D) mice exposed to cigarette smoke for 4 weeks and room air for 1 week (SC1w); (E) mice exposed to cigarette smoke for 4 weeks and room air for 4 weeks (SC4w). 1, Mac-3 antigen indicated as green fluorescence; 2, MMP-12 antigen indicated as red fluorescence; 3, nucleus staining indicated as blue fluorescence; 4, merged image, in which yellow fluorescence indicates the colocalization of MMP-12 antigen and macrophages. MMP, matrix metalloproteinase.

The protein expression of MMP-12 was upregulated in the lungs of the mice exposed to cigarette smoke for 4 weeks compared with the control mice (P= 0.014); however, no significant differences were observed between the mice exposed to cigarette smoke for 4 weeks and those exposed for 8 weeks (P=0.92). The protein expression levels of MMP-12 were significantly higher in the mice 1 week following smoking cessation compared with the control group (P=0.009; Fig. 3). Double immunofluorescence labeling demonstrated that exposure to cigarette smoke induced macrophages to secrete MMP-12 and increased their recruitment into the lung parenchyma and airways. However, macrophages, which secreted MMP-12 following smoking cessation were rarely seen in the lung parenchyma and were located predominantly around the bronchial walls (Fig. 4). MMP-12 antigens (red fluorescence) were located in the cytoplasm and Mac-3 (green fluorescence) was distributed predominantly in the cytomembrane and cytoplasm. DAPI staining (blue fluorescence) was used to stain the cell nuclei. In the three-channel composite images, yellow fluorescence represented the colocalization of MMP-12 and Mac-3.

### Expression and distribution of TGF-β1 protein

**Figure 5:**
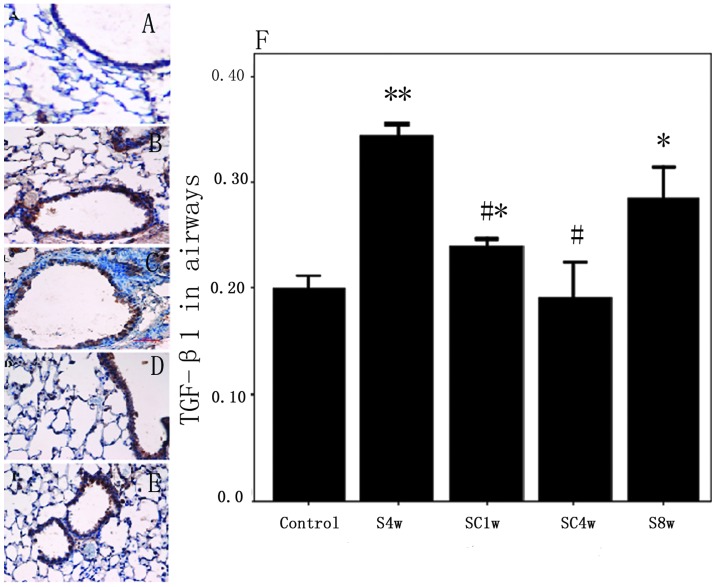
Immunohistochemical staining for TGF-β1 around the small airways of each group at 400x magnification. Brown indicates a positive result. (A) Control; (B) mice exposed to cigarette smoke for 4 weeks (S4w); (C) mice exposed to cigarette smoke for 8 weeks (S8w); (D) mice exposed to cigarette smoke for 4 weeks and room air for 1 week (SC1w); (E) mice exposed to cigarette smoke for 4 weeks and room air for 4 weeks (SC4w). (F) Comparison of the protein expression of TGF-β1 between the groups. Data are presented as the mean ± standard error. ^*^P<0.05 and ^**^P<0.001 vs. the control mice; ^#^P<0.001 vs. the S4w mice. TGF, transforming growth factor.

**Figure 6:**
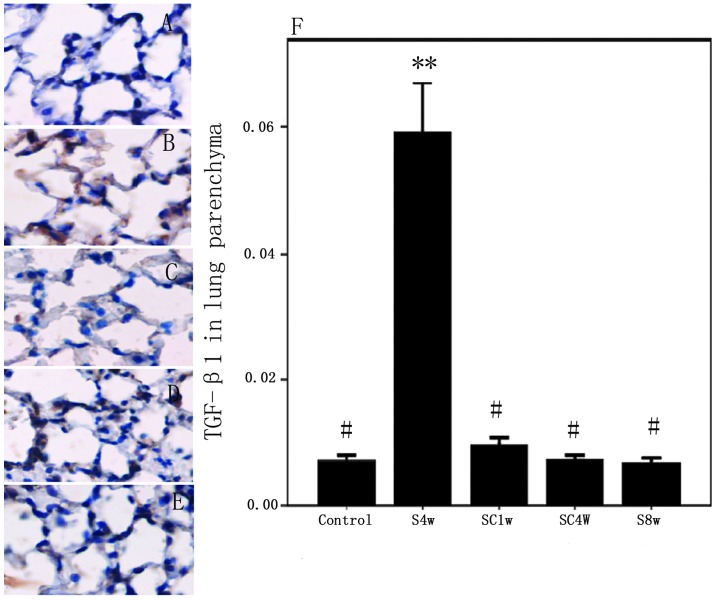
Immunohistochemical staining for TGF-β1 in the lung parenchyma from each group (magnification, x400). Brown indicates a positive result. (A) Control; (B) mice exposed to cigarette smoke for 4 weeks (S4w); (C) mice exposed to cigarette smoke for 8 weeks (S8w); (D) mice exposed to cigarette smoke for 4 weeks and room air for 1 week (SC1w); (E) mice exposed to cigarette smoke for 4 weeks and room air for 4 weeks (SC4w). (F) Protein expression of TGF-β1 between the groups. Data are presented as the mean ± standard error. ^**^P<0.001 vs. the control mice; ^#^P<0.001 vs. the S4w mice. TGF, transforming growth factor.

The protein expression of TGF-β1 was upregulated in the airways and lung parenchyma of the mice exposed to cigarette smoke for 4 weeks compared with the control mice (P<0.001). No further increase was observed in the protein expression of TGF-β1 in the airways (P=0.077), but the levels were significantly decreased in the lung parenchyma (P=0.000) of the mice exposed to cigarette smoke for 8 weeks compared with those exposed for 4 weeks. Following smoking cessation for 4 weeks and 1 week, respectively, the protein expression levels of TGF-β1 were returned to those observed in the normal control group in the airways and lung parenchyma (Figs. 5 and 6).

## Discussion

COPD is a chronic inflammatory airway disease, which is characterized by airflow limitations that are not fully reversible. The typical pathological changes associated with COPD are enlargement of the alveolar airspaces and remodeling of the small airways, which are closely associated with airflow obstruction (2–4). Smoking cessation is an effective way of relieving the inflammation and airflow obstruction associated with COPD, and has been advocated worldwide (1). However, investigation of the benefits of smoking cessation on lung tissue is predominantly based on patients in whom COPD has developed (18,17). Therefore, the impact of smoking cessation on the inflammatory response and lung lesions prior to the establishment of typical pathological changes remains to be elucidated.

Establishment of emphysema in the majority of murine models requires exposure to cigarette smoke for ≥24 weeks (14,15). The present study established a group of mice subjected to subacute exposure to cigarette smoke for 4 weeks (18), an early cessation group, in which smoking was terminated after 4 weeks, and control groups, in which the mice were exposed to filtered air for 4 weeks and to cigarette smoke for 8 weeks. These groups were established to observe the impact of early cessation of smoking on the pathological changes and inflammatory responses in the lung.

AMs are considered the main inflammatory cells of the lower respiratory tract and the first line of defense against invasion of the lungs by foreign microorganisms (19,20). AMs ingest, transport and remove exogenous particulate substances and pathogenic microorganisms through phagocytosis, pinocytosis and digestion. AMs are also involved in antigen presentation and the regulation of inflammatory reactions (19,21–23).

There is evidence that AMs activate and release proteolytic enzymes, an action that is closely associated with the development of emphysema (19,24,21). There is also a correlation between the severity of COPD and the number of AMs (20,25,26). MMP-12 is predominantly derived from AMs. MMP-12 was first identified in the AMs of smokers (27), and degrades the protein components of the extracellular matrix (ECM), including elastin, fibronectin, laminin and gelatin (28,29). Furthermore, MMP-12 is involved in the metabolism, cell migration, tissue repair and remodeling of the ECM and high expression levels of MMP-12 can lead to degradation of pathological proteins in the ECM (30). Previous studies have demonstrated that MMP-12 is important in the establishment of a cigarette smoke-induced murine model of emphysema, and that MMP-12 gene knockout can result in the avoidance of emphysema (19,28).

Quantitative analyses of the size of alveolar airspaces in experimental animal models usually involve the mean linear intercept (31–33). However, due to the heterogeneity of pulmonary lesions and the early stage of lung damage, the mean linear intercept may not be sufficiently sensitive to reveal localized enlargements of alveolar airspaces (14). The present study calculated the sizes of the alveolar airspaces at different distances from the terminal bronchiole, according to the method of Biselli *et al* (14). The present study observed, similar to Biselli *et al*, that the alveolar septum 100 *μ*m from the terminal bronchiole was enlarged in mice following subacute exposure to cigarette smoke. Furthermore, infiltration of AMs was increased and the protein expression of MMP-12 was upregulated. Increased infiltration of AMs during exposure to cigarette smoke is a normal defense mechanism of the body, but an excessive increase and release of proteolytic enzymes induces local damage to the lung parenchyma (19,20,24,25). However, the alveolar airspaces, pulmonary infiltration of AMs and protein expression of MMP-12 were not significantly different between the 8 and 4 week smoke-exposure groups. Therefore, the inflammatory response appeared to decrease over time. Similar to emphysema, locally enlarged alveolar airspaces did not recover following smoking cessation and, although the infiltration of AMs and expression of MMP-12 improved, they remained higher compared with the normal control group. It has been reported that degraded fragments of elastin are a powerful type of chemokine, which recruits AMs to lesions (34). Immunofluorescent localization demonstrated that, following smoking cessation, the AMs secreting MMP-12 were predominantly distributed in the walls of the small airways. However, the correlation between the expression of MMP-12 and the generation of locally degraded fragments of elastin requires further investigation.

Remodeling of the small airways is associated with damage and abnormal repair of the ECM (35,36), however, the pathogenesis remains to be elucidated. TGF-β1 promotes tissue repair by improving the expression of protein components of the ECM, including collagen and fibronectin, and inhibiting degradation of the ECM (37). MMP-12 degrades the ECM or inhibits TGF-β1 directly, indirectly downregulates the synthesis of the ECM (30) and prevents excessive repair of fibrosis. Interactions between TGF-β1 and MMP-12 are closely associated with the remodeling of small airways (30). Cigarette smoke-induced inflammation has previously been shown to promote the production and release of TGF-β1, and the expression of TGF-β1 is increased in the airway epithelial cells from smokers and patients with COPD (35). Increased expression of TGF-β1 have been correlated with the dysfunction of bronchial basal membranes and the number of peribronchiolar fibroblasts (38). The present study demonstrated that the expression levels of TGF-β1 in the small airways and lung parenchyma were upregulated after 4 weeks of smoking exposure and returned to normal 1 week after smoking cessation; however, abnormally increased collagen deposition was not observed, which may be due to the early stage of disease or upregulation in the expression of MMP-12. Compared with the mice exposed to cigarette smoke for 4 weeks, the expression levels of TGF-β1 in the small airways of the mice exposed to cigarette smoke for 8 weeks exhibited no further increase. Furthermore, no abnormally increased deposition of collagen around the small airways was observed and the expression of TGF-β1 in the lung parenchyma was decreased. These results are concordant with those of Churg *et al* (36), which suggested that distinctly different mechanisms regulate ECM metabolism in the airway wall and lung parenchyma. High expression levels of TGF-β1 and abnormal repair may lead to remodeling of the small airways and airway obstruction, whereas reduced expression levels of TGF-β1 and insufficient repair of tissue may contribute to the development of emphysema. Therefore, TGF-β1 is an important regulator of inflammation and remodeling, and may offer a novel potential therapeutic strategy for COPD.

In conclusion, the inflammatory reaction and damage to the lung parenchyma, induced by subacute exposure to cigarette smoke, occurred prior to remodeling of the walls of the small airways. Lung tissue has a self-limiting ability in response to chronic exposure to smoke, and the pulmonary inflammatory reaction and pathological damage were not increased by prolonging the exposure to cigarette smoke for a certain period of time. Local damage to the lung parenchyma was not reversed by early cessation of smoking, whereas the pulmonary inflammatory response was partially downregulated. In addition, macrophage infiltration and the expression of MMP-12 did not return to normal following smoking cessation. Following smoking cessation, the expression of MMP-12 was distributed predominantly in the airways, which may prevent the excessive deposition of collagen or may be due to excessive locally degraded fragments of elastin. Regulation of the expression levels of MMP-12 and TGF-β1, particularly regulation of the distribution in the airways and lung parenchyma, may be a potential strategy for the early treatment of COPD.

## Figures and Tables

**Table I:** Comparison of the number of alveolar airspaces at a distance of ~100 *μ*m from the terminal bronchioles in each group.

Group	Number	Mean	STD
Control	5	25.60	1.97
S4w	5	31.62^b^	2.65
SC1w	5	29.24^b^	1.42
SC4w	5	29.88^a^	2.66
S8w	5	32.94^b^	2.74

S4w, mice exposed to cigarette smoke for 4 weeks; SC1w, mice exposed to cigarette smoke for 4 weeks followed by 1 week exposure to room air; SC4w, mice exposed to cigarette smoke for 4 weeks followed by 4 weeks exposure to room air; S8w, mice exposed to cigarette smoke for 8 weeks; STD, standard deviation.

a P<0.05 vs. the control mice;

b P<0.01 vs. the control mice.

## References

[1] Vestbo J, Hurd SS, Agusti AG (2013). Global strategy for the diagnosis, management, and prevention of chronic obstructive pulmonary disease: GOLD executive summary. Am J Respir Crit Care Med.

[2] Wright JL, Postma DS, Kerstjens HA, Timens W, Whittaker P, Churg A (2007). Airway remodeling in the smoke exposed guinea pig model. Inhal Toxicol.

[3] Cosio M, Ghezzo H, Hogg JC (1978). The relations between structural changes in small airways and pulmonary-function tests. N Engl J Med.

[4] Hasegawa M, Nasuhara Y, Onodera Y (2006). Airflow limitation and airway dimensions in chronic obstructive pulmonary disease. Am J Respir Crit Care Med.

[5] D’hulst AI, Vermaelen KY, Brusselle GG (2005). Time course of cigarette smoke-induced pulmonary inflammation in mice. Eur Respir J.

[6] Simmons MS, Connett JE, Nides MA (2005). Smoking reduction and the rate of decline in FEV(1): results from the Lung Health Study. Eur Respir J.

[7] Willemse BW, Postma DS, Timens W, ten Hacken NH (2004). The impact of smoking cessation on respiratory symptoms, lung function, airway hyperresponsiveness and inflammation. Eur Respir J.

[8] Willemse BW, ten Hacken NH, Rutgers B, Lesman-Leegte IG, Postma DS, Timens W (2005). Effect of 1-year smoking cessation on airway inflammation in COPD and asymptomatic smokers. Eur Respir J.

[9] Gamble E, Grootendorst DC, Hattotuwa K (2007). Airway mucosal inflammation in COPD is similar in smokers and ex-smokers: a pooled analysis. Eur Respir J.

[10] Lapperre TS, Postma DS, Gosman MM (2006). Relation between duration of smoking cessation and bronchial inflammation in COPD. Thorax.

[11] Braber S, Henricks PA, Nijkamp FP, Kraneveld AD, Folkerts G (2010). Inflammatory changes in the airways of mice caused by cigarette smoke exposure are only partially reversed after smoking cessation. Respir Res.

[12] Wang Z, Zhang JN, Hu XF (2010). Effects of pentoxifylline on Wnt/β-catenin signaling in mice chronically exposed to cigarette smoke. Chin Med J (Engl).

[13] Zhang JN, Wang Z, Shi W (2009). Mechanism responsible for pu lmonary fibrosis induced by concomitant chronic smoke exposure and pentoxifylline administration. Chin J Pathophysiol (Chin).

[14] Biselli PJ, Lopes FD, Moriya HT (2011). Short-term exposure of mice to cigarette smoke and/or residual oil fly ash produces proximal airspace enlargments and airway epithelium remodeling. Braz J Med Biol Res.

[15] Bracke KR, Dentener MA, Papakonstantinou E (2010). Enhanced deposition of low-molecular-weight hyaluronan in lungs of cigarette smoke-exposed mice. Am J Respir Cell Mol Biol.

[16] Tønnesen P, Carrozzi L, Fagerström K (2007). Smoking cessation in patients with respiratory diseases: a high priority, integral component of therapy. Eur Respir J.

[17] Kotz D, Wesseling G, Huibers MJ, van Schayck OC (2009). Efficacy of confronting smokers with airflow limitation for smoking cessation. Eur Respir J.

[18] Demoor T, Bracke KR, Dupont LL (2011). The role of ChemR23 in the induction and resolution of cigarette smoke-induced inflammation. J Immunol.

[19] Hautamaki RD, Kobayashi DK, Senior RM, Shapiro SD (1997). Requirement for macrophage elastase for cigarette smoke-induced emphysema in mice. Science.

[20] Shapiro SD (1999). The macrophage in chronic obstructive pulmonary disease. Am J Respir Crit Care Med.

[21] Bitterman PB, Wewers MD, Rennard SI, Adelberg S, Crystal RG (1986). Modulation of alveolar macrophage-driven fibroblast proliferation by alternative macrophage mediators. J Clin Invest.

[22] Houghton AM, Quintero PA, Perkins DL (2006). Elastin fragments drive disease progression in a murine model of emphysema. J Clin Invest.

[23] Venet A, Hance AJ, Saltini C, Robinson BW, Crystal RG (1985). Enhanced alveolar macrophage-mediated antigen-induced T-lymphocyte proliferation in sarcoidosis. J Clin Invest.

[24] Shapiro SD, Ingenito EP (2005). The pathogenesis of chronic obstructive pulmonary disease: advances in the past 100 years. Am J Respir Cell Mol Biol.

[25] Hogg JC, Chu F, Utokaparch S (2004). The nature of small-airway obstruction in chronic obstructive pulmonary disease. N Engl J ed.

[26] Finkelstein R, Fraser RS, Ghezzo H, Cosio MG (1995). Alveolar inflammation and its relation to emphysema in smokers. Am J Respir Crit Care Med.

[27] Shapiro SD, Kobayashi DK, Ley TJ (1993). Cloning and characterization of a unique elastolytic metalloproteinase produced by human alveolar macrophages. J Biol Chem.

[28] Chandler S, Cossins J, Lury J, Wells G (1996). Macrophage metalloelastase degrades matrix and myelin proteins and processes a tumour necrosis factor-alpha fusion protein. Biochem Biophys Res Commun.

[29] Gronski TJ, Martin RL, Kobayashi DK (1997). Hydrolysis of a broad spectrum of extracellular matrix proteins by human macrophage elastase. J Biol Chem.

[30] England KA, Price AP, Tram KV, Shapiro SD, Blazar BR, Panoskaltsis-Mortari A (2011). Evidence for early fibrosis and increased airway resistance in bone marrow transplant recipient mice deficient in MMP12. Am J Physiol Lung Cell Mol Physiol.

[31] Thurlbeck WM, Galaugher W, Mathers J (1981). Adaptive response to pneumonectomy in puppies. Thorax.

[32] Yao H, Chung S, Hwang JW (2012). SIRT1 protects against emphysema via FOXO3-mediated reduction of premature senescence in mice. J Clin Invest.

[33] Podowski M, Calvi C, Metzger S (2012). Angiotensin receptor blockade attenuates cigarette smoke-induced lung injury and rescues lung architecture in mice. J Clin Invest.

[34] Houghton AM, Quintero PA, Perkins DL (2006). Elastin fragments drive disease progression in a murine model of emphysema. J Clin Invest.

[35] Takizawa H, Tanaka M, Takami K (2001). Increased expression of transforming growth factor-beta1 in small airway epithelium from tobacco smokers and patients with chronic obstructive pulmonary disease (COPD). Am J Respir Crit Care Med.

[36] Churg A, Tai H, Coulthard T, Wang R, Wright JL (2006). Cigarette smoke drives small airway remodeling by induction of growth factors in the airway wall. Am J Respir Crit Care Med.

[37] Philipp K, Riedel F, Germann G, Hörmann K, Sauerbier M (2005). TGF-beta antisense oligonucleotides reduce mRNA expression of matrix metalloproteinases in cultured wound-healing-related cells. Int J Mol Med.

[38] Königshoff M, Kneidinger N, Eickelberg O (2009). TGF-beta signaling in COPD: deciphering genetic and cellular susceptibilities for future therapeutic regimen. Swiss Med Wkly.

